# Chronic shoulder injury related to vaccine administration following coronavirus disease 2019 vaccination: a case report

**DOI:** 10.1186/s13256-023-04198-0

**Published:** 2023-10-17

**Authors:** Masahiro Miyano, Yukinori Tsukuda, Shigeto Hiratsuka, Masanari Hamasaki, Norimasa Iwasaki

**Affiliations:** 1 Department of Orthopaedic Surgery, Otaru General Hospital, Wakamatsu 1-1-1, Otaru, Hokkaido 047-8550 Japan; 2 Department of Orthopaedic Surgery, Faculty of Medicine and Graduate School of Medicine, Hokkaido University, Sapporo, Japan

**Keywords:** Shoulder injury related to vaccine administration (SIRVA), COVID-19, Vaccine, Subacromial–subdeltoid bursitis, Intramuscular injection

## Abstract

**Background:**

Shoulder injury related to vaccine administration, defined as shoulder pain and limited range of motion occurring after administration in the upper arm, has been previously reported. The symptom resolved completely after treatment with oral nonsteroidal anti-inflammatory drugs or an intraarticular steroid injection, however there have been few reports of long-term symptoms following coronavirus disease 2019 vaccination. This case report describes a healthy, middle-aged, healthcare worker who developed post-vaccination subacromial–subdeltoid bursitis that lasted for more than 6 months after Pfizer–BioNTech coronavirus disease 2019 vaccination.

**Case presentation:**

A 55-year-old Japanese woman with no significant medical history was vaccinated in the standard site, with the needle direction perpendicular to the skin. Within a few hours after the second vaccination, severe shoulder pain and limited range of motion appeared. Although shoulder range of motion improved, her shoulder pain did not improved for several months, and she consulted an orthopedic doctor 5 months later. Radiographs of her left shoulder did not provide helpful diagnostic information. High intensity in the subacromial–subdeltoid space was seen on short TI inversion recovery of magnetic resonance imaging, showing subacromial–subdeltoid bursitis. She was diagnosed with a shoulder injury related to vaccine administration. The patient was started on an oral anti-inflammatory drug, and the left subacromial space was injected with 2.5 mg of betamethasone with 3 ml of 1% lidocaine without epinephrine every 2 weeks. One month after starting this treatment, since her shoulder pain had not improved, the oral anti-inflammatory drug was switched to tramadol hydrochloride acetaminophen. However, 3 months after switching medication, the shoulder pain continued, and she worked so as to have minimal impact on her shoulder.

**Conclusion:**

A case of subacromial–subdeltoid bursitis following a second dose of the Pfizer–BioNTech coronavirus disease 2019 vaccine that lasted many months is reported. Injection technique is a modifiable risk factor, the adverse effects of which could potentially be mitigated with appropriate and relevant training of healthcare providers. To prevent this type of case, the appropriate landmark, needle length, and direction should be confirmed.

## Background

After any vaccination, adverse effects may occur, and they can be divided into systemic and local effects. Systemic adverse effects include headache, fatigue, chills, fever, nausea, diarrhea, muscle pain, and joint pain, whereas local effects include pain, redness, swelling, itching, and tenderness [1]. These symptoms are usually mild and transient and resolve within 1 week after the injection [2–4]. Shoulder injury related to vaccine administration (SIRVA), defined as shoulder pain and limited range of motion (ROM) occurring after administration in the upper arm, has been previously reported [5–7]. Prevalence is unknown. The pathogenesis of this condition is an immune response to intraarticular inoculation after vaccination [3], which is usually treated with medications such as oral nonsteroidal anti-inflammatory drugs or intraarticular steroid injection [5, 8]. SIRVA usually occurs due to incorrect intramuscular injection technique, such as inappropriate needle direction or depth of penetration. SIRVA includes a variety of conditions such as shoulder periarthritis, tendinopathy, and subacromial–subdeltoid bursitis, and diagnosis of this condition can be challenging, because other pathologies, including subacromial bursitis, rotator cuff tendinopathy, rotator cuff tear, or adhesive capsulitis, overlap significantly with the symptomatology of SIRVA [3, 9].

Vaccination for coronavirus disease-2019 (COVID-19) is no exception, in that there are various adverse symptoms of SIRVA [10–14]. Many doses of COVID-19 vaccinations have been administered around the world during the COVID-19 pandemic, although there are still people who have not been vaccinated because of these harmful effects. Therefore, the healthcare community needs to investigate how many adverse effects there are and provide correct information to patients. There are a few previous case reports about SIRVA due to COVID-19 vaccination. Most of the literature reported subacromial–subdeltoid bursitis, such as shoulder periarthritis, and the symptoms resolved completely after treatment with oral nonsteroidal anti-inflammatory drugs or an intraarticular steroid injection [10–14]. However, there have been few reports of long-term symptoms following COVID-19 vaccination. This case report describes a healthy, middle-aged, healthcare worker who developed postvaccination subacromial–subdeltoid bursitis that lasted for more than 6 months after the Pfizer–BioNTech COVID-19 vaccination. Each day, many people are given this vaccination worldwide, so this case follows that the number of cases of SIRVA due to inappropriately injected vaccines may also be increasing.

## Case presentation

**Fig. 1:**
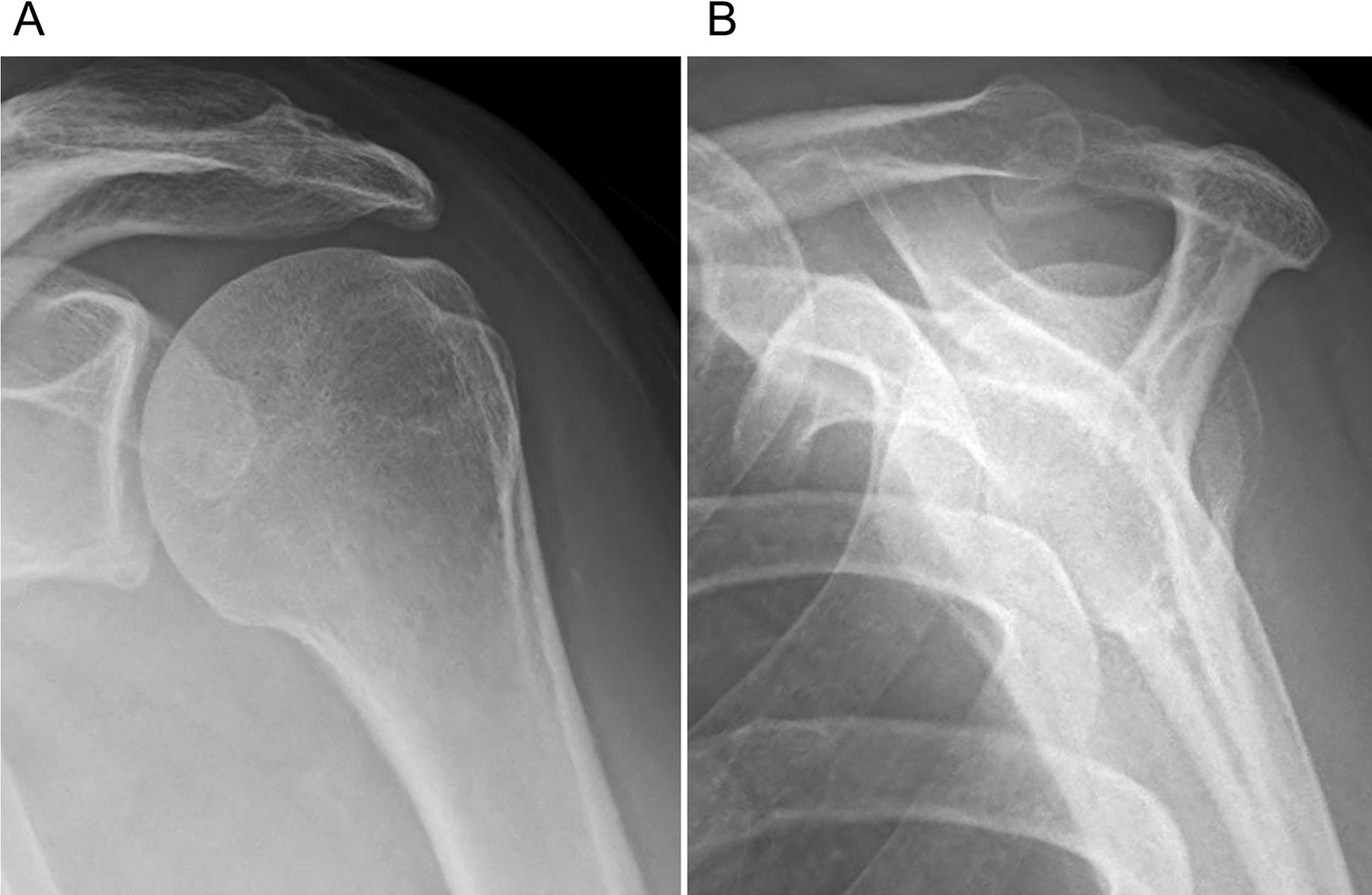
Radiographs of the left shoulder at the initial visit. **A** Anteroposterior view, **B** scapular Y view

**Fig. 2:**
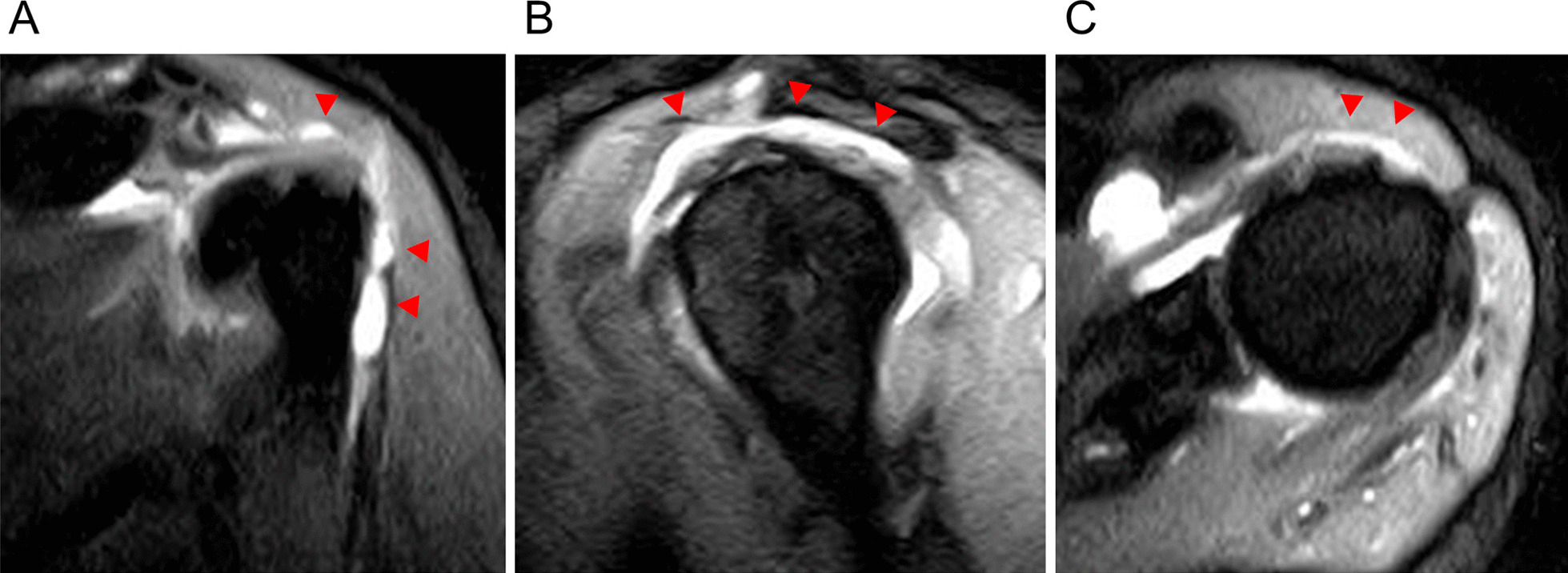
Short TI inversion recovery of magnetic resonance imaging of the left shoulder (initial visit). **A** Coronal view, **B** sagittal view. **C** axial view. Images 5 months after receiving the COVID-19 vaccine demonstrate effusion in the subacromial–subdeltoid bursa suggesting bursitis (arrowheads)

A 55-year-old Japanese woman with no significant medical history received a Pfizer–BioNTech COVID-19 vaccination using a 25-gauge needle, 1 inch in length, in an injection site based on the landmark of three finger-breadths below the midlateral border of the acromial process. The needle direction was perpendicular to the skin. Within a few hours after injection of this vaccine, severe shoulder pain (visual analogue scale 10/10) and limited ROM appeared. Although the restricted ROM of her shoulder improved, her shoulder pain was not improved after several months. Therefore, she decided to see an orthopedic doctor after 5 months, as her work had prevented her from seeing the doctor sooner. Radiographs (anteroposterior, scapula Y) of her left shoulder were performed and did not provide helpful diagnostic information (Fig. 1). High intensity in the subacromial–subdeltoid space was observed on short TI inversion recovery (STIR) of magnetic resonance imaging (MRI), which was considered to show subacromial–subdeltoid bursitis (Fig. 2). She was diagnosed SIRVA. The patient was started on an oral anti-inflammatory drug, and the left subacromial space was injected with 2.5 mg betamethasone with 3 ml of 1% lidocaine without epinephrine every two weeks. One month after starting this treatment, her shoulder pain had not improved, so the oral anti-inflammatory drug was switched to tramadol hydrochloride acetaminophen. Physical therapy was not possible because of her work. However, 3 months after switching medication, the shoulder pain remained, and began interfering with her work.

## Discussion and conclusion

SIRVA is a rare condition that occurs within 48 hours following vaccine injection, especially influenza vaccination [5–8]. Atanasoff *et al.* reported that 93% of patients had pain onset within 24 hours of vaccination, with 54% reporting immediate pain [3]. Most cases occurred as a result of the penetration of the needle into the subacromial–subdeltoid space by inappropriate intramuscular injection technique, and, as a consequence, shoulder pain or restricted ROM developed after vaccination. The differential diagnosis of SIRVA includes shoulder periarthritis and tendinopathy, but the criteria for a definitive diagnosis of SIRVA are unclear. Therefore, SIRVA is diagnosed base on the patient’s clinical course. Whereas most of these reactions are mild and transient, they may rarely persist and impact quality of life significantly [15]. SIRVA is one such condition that can lead to persistent musculoskeletal dysfunction. The exact prevalence and incidence of SIRVA are unclear. In a previous report, Martin Arias *et al. * found 45 cases of new-onset, unilateral shoulder dysfunction following vaccine administration [2]. Most of the cases occurred following influenza vaccine, the second most frequent vaccine was pneumococcal vaccine, and shoulder disability was also reported after tetanus–diphtheria toxoid, human papilloma virus, and hepatitis A virus vaccines [2, 3]. From these reports, it can be seen that SIRVA has the potential to occur following various vaccines. One of the reasons that most of the cases have been observed following influenza vaccination is the fact that it is administered to the largest patient population. Therefore, cases of similar symptoms may also be increasing after COVID-19 vaccination, because of the massive promotion of COVID-19 vaccination worldwide.

Recently, similar outcomes have been reported with the COVID-19 vaccine [10–13]. Wharton *et al.* reported a young man who developed acute onset of left shoulder pain, weakness, and stiffness 24 hours after receiving his second dose of the Moderna COVID-19 vaccine. His left subacromial space was injected with 20 mg of triamcinolone with 9 cc of 1% lidocaine without epinephrine 2.5 weeks after the vaccination, and, 6 days after this treatment, the patient reported complete resolution of the pain [10]. Boonsri *et al.* reported that a middle-aged woman presented with severe right shoulder pain about 3 hours after receiving a second shot of the AstraZeneca COVID-19 vaccine; subacromial–subdeltoid bursitis and a supraspinatus tendon tear were observed on ultrasonography, and as a consequence, her shoulder pain improved after treatment with an oral nonsteroidal anti-inflammatory drug [11]. Furthermore, similar phenomena were reported after administration of other COVID-19 vaccines manufactured by Pfizer–BioNTech and Sinovac [12, 13]. Given these reports, COVID-19 vaccines are also seen as a cause of SIRVA, and the symptoms of SIRVA after COVID-19 vaccine continued for a short term, usually less than 1 week.

Although very uncommon, SIRVA lasting many months was reported previously after another vaccination [15]. In the present case, SIRVA lasted for more than 6 months and occurred after administration of the COVID-19 vaccine. The first reason for the long duration of inflammation in the present patient may be the preexistence of asymptomatic shoulder periarthritis, and degeneration of the shoulder joint may have become symptomatic because of the vaccination. Prior to the administration of the vaccine, the subject should be carefully asked about the presence of shoulder joint disease. The second reason may be that this patient did not receive suitable treatment immediately. The recommended treatments for SIRVA in the acute phase include nonsteroidal anti-inflammatory drugs, physical therapy, and intraarticular corticosteroid injections [16]. In the present case, the patient was treated after 5 months, so she might have missed the optimal period for treatment of SIRVA. The third reason may be a strong immune response because of repeated vaccine administration. Atanasoff *et al.* suggested that injecting vaccine antigens into people who have been sensitized due to a previous vaccination may increase their susceptibility to SIRVA [3]. Moreover, preexisting antibodies in the subdeltoid bursa due to previous vaccination may prolong the inflammatory response in the case of administration of a vaccine into the subdeltoid bursa [17]. Sahin *et al.* suggested that the Pfizer–BioNTech BNT162b2 mRNA COVID-19 vaccine elicited high severe acute respiratory syndrome coronavirus 2 (SARS-CoV-2) neutralizing antibody titers and robust antigen specific T cell responses [18]. In addition, this vaccine may be highly immunogenic, which can affect the reactogenicity and the side-effect profile, and stronger inflammatory reactions were observed after the second rather than after the first vaccination [19]. If the injection is administered into the subdeltoid space, a strong inflammatory reaction may be prone to occur there. Thus, in the case of COVID-19 vaccination, repeated vaccine administration may have resulted in a higher rate of SIRVA for a long time due to a strong inflammatory effect. To the best of our knowledge, there are no previous reports regarding SIRVA continuing for months after COVID-19 vaccination. Administration of COVID-19 vaccinations will continue worldwide because COVID-19 vaccination is one of the important preventive measures for SARS-CoV-2 infection, so long-lasting SIRVA may be increase in parallel. Long-lasting SIRVA affects quality of life significantly [15], therefore, we need to take countermeasures against long-lasting SIRVA. However, the mechanism of long-lasting SIRVA remains unknown, and its pathological mechanism needs to be clarified.

The recommended intramuscular injection site and the direction of the needle at the deltoid are three finger-breadths below the midlateral border of the acromial process and perpendicular to the skin, respectively. In the present case, the patient was injected using a 1-inch, 25-gauge needle with the entry point three finger-breadths below the midlateral border of the acromial process with the direction of the needle perpendicular to the skin. This distance from the midlateral border of the acromial process to the entry point and the direction of the needle were correct, but SIRVA nevertheless occurred. One reason for the SIRVA could be penetration of the needle deeper than the thickness of the deltoid. Boder *et al.* reported that the extent of the subdeltoid bursa from the acromion ranged from 30 to 60 mm, and that the depth from the skin to the subdeltoid bursa was between 8 and 16 mm [20]. Therefore, the needle may enter the subdeltoid space even if this vaccination is administered at the right location three finger-breadths (about 30–40 mm) below the midlateral border of the acromial process. Furthermore, a 1-inch (25.4 mm) needle may have the potential to enter the subdeltoid space by penetrating the deltoid. We note that the present patient was not particularly thin, with a BMI of 26.5 kg/m^2^, as it is important to take into account an individual’s muscle size when performing intramuscular injection of the deltoid by adjusting needle size. 

SIRVA is not widely recognized in Japan. One reason may be that subcutaneous injections are recommended. Vaccines, such as influenza and pneumococcal vaccines, are injected intramuscularly in most countries, but intramuscular injections tend to be avoided in Japan. Another reason may be that vaccines were administered at the humerus, not the shoulder, level. Thus, SIRVA has rarely happened in Japan before now. The COVID-19 vaccine is basically injected intramuscularly. In addition, administration of these vaccines will continue because COVID-19 vaccination is one of the important preventive measures for SARS-CoV-2 infection. Therefore, recognition of SIRVA must be increased, especially in countries such as Japan that recommend subcutaneous injections.

A case of subacromial–subdeltoid bursitis following a second dose of the Pfizer–BioNTech COVID-19 vaccine that lasted many months was reported. To the best of our knowledge, this is the first report of post-vaccination subacromial–subdeltoid bursitis lasting many months after COVID-19 vaccination. To prevent this type of case, the appropriate landmark, needle length, and needle direction should be confirmed. In cases of repeated administrations per person, healthcare workers should keep this adverse effect in mind when injecting this vaccine.

## Data Availability

The datasets used and analyzed during the current study are available from the corresponding author on reasonable request.

## Abbreviations

SIRVA: Shoulder injury related to vaccine administration
ROM: Range of motion
COVID-19: Coronavirus disease-2019
